# Urological complication following aortoiliac graft: case report and review of the literature

**DOI:** 10.1590/S1516-31802010000300010

**Published:** 2010-05-06

**Authors:** Leonardo Lima Borges, Fábio César Miranda Torricelli, Gustavo Xavier Ebaid, Antônio Marmo Lucon, Miguel Srougi

**Affiliations:** I MD. Resident, Department of Urology, Faculdade de Medicina da Universidade de São Paulo (FMUSP), São Paulo, Brazil.; II MD. Urological surgeon, Department of Urology, Faculdade de Medicina da Universidade de São Paulo (FMUSP), São Paulo, Brazil.; III MD, PhD. Urological surgeon, Department of Urology, Faculdade de Medicina da Universidade de São Paulo (FMUSP), São Paulo, Brazil.; IV MD, PhD. Full professor and chairman, Department of Urology, Faculdade de Medicina da Universidade de São Paulo (FMUSP), São Paulo, Brazil.

**Keywords:** Ureter, Ureteral obstruction, Kidney, Hydronephrosis, Blood vessel prosthesis, Ureter, Obstrução ureteral, Rim, Hidronefrose, Prótese vascular

## Abstract

**CONTEXT:**

Ureteral stenosis and ureterohydronephrosis may be serious complications of aortoiliac or aortofemoral reconstructive surgery.

**CASE REPORT:**

A 62-year-old female patient presented with a six-month history of left lumbar pain. She was a smoker, and had mild chronic arterial hypertension and Takayasu arteritis. She had previously undergone three vascular interventions. In two procedures, Dacron prostheses were necessary. Excretory urography showed moderate left ureterohydronephrosis and revealed a filling defect in the ureter close to where the iliac vessels cross. This finding was compatible with ureteral stenosis, and the aortoiliac graft may have been the reason for this inflammatory process. The patient underwent laparotomy, which showed that there was a relationship between the ureteral stenosis and the vascular prosthesis. Segmental ureterectomy and end-to-end ureteroplasty with the ureter crossing over the prosthesis anteriorly were performed. There were no complications. The early and late postoperative periods were uneventful. The patient evolved well and the results from a new excretory urogram were normal. We concluded that symptomatic ureterohydronephrosis following aortoiliac graft is a real complication and needs to be quickly diagnosed and treated by urologists.

## RESUMO

**INTRODUÇÃO:**

Estenose ureteral e ureterohidronefrose podem ser sérias complicações da cirurgia reconstrutiva aorto-femoral ou aorto-ilíaca.

**RELATO DE CASO:**

Uma paciente de 62 anos apresentou-se referindo história de dor lombar a esquerda há seis meses. Ela era fumante, portadora de hipertensão arterial crônica leve e arterite de Takayasu. Havia sido submetida a três intervenções vasculares. Em dois procedimentos o uso de prótese de Dacron foi necessário. Uma urografia excretora revelou moderada ureterohidronefrose à esquerda e falha de enchimento no ureter próximo ao cruzamento dos vasos ilíacos. Esse achado era compatível com uma estenose ureteral e o enxerto aorto-ilíaco poderia ser a causa do processo inflamatório. A paciente foi submetida a laparotomia, que evidenciou a relação entre estenose ureteral e a prótese vascular. Ureterectomia segmentar e uretroplastia término-terminal com o ureter passando anteriormente à prótese foram realizadas. Não ocorreram complicações. Os períodos de pós-operatório precoce e tardio transcorreram sem intercorrências. A paciente evoluiu bem e uma nova urografia excretora apresenta-se normal. Concluímos que ureterohidronefrose sintomática após enxerto aorto-ilíaco é uma complicação real e precisa ser rapidamente diagnosticada e tratada pelo urologista.

## INTRODUCTION

Ureteral stenosis and ureterohydronephrosis may be serious complications in aortoiliac or aortofemoral reconstructive surgery. Ureteral lesions from vascular surgery are believed to account for 0.8% of lesions recognized at the time of surgery and 2.2% of complications observed later.^1^ Ureteral lesions are sometimes not iatrogenic. They are related to the aneurysmal form of aortic disease, and some develop from inflammatory secondary reactions. Today, intraoperative ureteral injury, secondary retroperitoneal fibrosis, residual hematomas, false aneurysms after surgery, graft placement anterior to the ureter and graft infection are the main causes of urological complications following vascular reconstructive surgery.^1^

We report on a case of a late complication from an aortoiliac graft in a symptomatic patient that needed surgical intervention. We also present a brief review of the literature on this subject.

## CASE REPORT

A 62-year-old Caucasian female presented with a six-month history of left lumbar pain. She had no history of fever, hematuria, dysuria, frequency, urgency, polyuria, nocturia, incontinence or difficulty in voiding. The patient was a smoker, and she had mild chronic arterial hypertension and Takayasu arteritis. Both of these diseases were under control with medications. She had previously undergone three vascular interventions because of complications from the Takayasu arteritis. In 1984, she underwent superior mesenteric artery endarterectomy. In 1986, she underwent aortobicarotid graft surgery with implantation of a Dacron prosthesis and in 1991, she underwent aortobiiliac graft surgery, also with a Dacron prosthesis.

Physical examination revealed only mild pain in the left flank. The patient was negative for Giordano’s sign. Urine culture and urine cytological tests were negative. Biochemical parameters were within normal limits. Abdominal ultrasonography (US) revealed a cyst in the left kidney. Abdominal computerized tomography (CT) just confirmed the diagnosis. The patient was submitted to US-guided aspiration puncture, follow by ethanol sclerosis.

**Figure 1.**
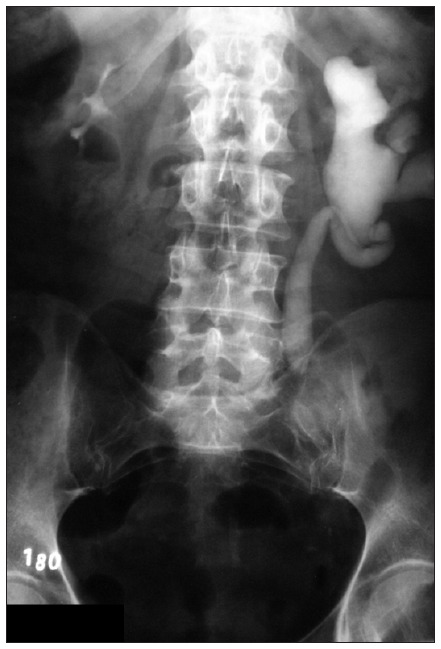
Intravenous urography showing moderate left ureterohydronephrosis and a filling defect in the ureter close to where the iliac vessels cross.

The pain kept bothering the patient and CT was performed again. This showed moderate left ureterohydronephrosis and a stop point in the mid-portion of the left ureter. Intravenous urography confirmed the moderate left ureterohydronephrosis and showed a filling defect in the ureter close to where the iliac vessels crossed (Figure 1). This was compatible with ureter stenosis, and the aortoiliac prosthesis may have been the reason for this inflammatory process. The patient underwent laparotomy, which showed that there was a relationship between the ureteral stenosis and the vascular prosthesis. Segmental ureterectomy and end-to-end ureteroplasty with the ureter crossing over the prosthesis were performed. Some fat tissue was interposed between the ureter and prosthesis and a double-J catheter was implanted.

There were no complications. The early and late postoperative periods were uneventful. The results from a new intravenous urogram were normal, i.e. contrast was eliminated through the ureter without filling defects. At a 24-month follow-up, the patient remained asymptomatic.

## DISCUSSION

The first case of hydronephrosis secondary to placement of an aortic prosthesis was reported by Jacobson et al. in 1962.^2^ Since then, a few other cases have been reported in the literature. We performed a search in the PubMed, Embase (Excerpta Medica), Cochrane library and Lilacs (Literatura Latino-Americana e do Caribe em Ciências da Saúde) databases (Table 1) and found 13 case reports and five case series with no more than three cases each, which reported occurrences of obstructive uropathy following vascular graft placement. Cases with ureteral fistula were not included.

**Table 1.** Search for case reports, in Medline (Medical Literature Analysis and Retrieval System Online), Embase (Excerpta Medica), Lilacs (Literatura Latino-Americana e do Caribe em Ciências da Saúde) and Cochrane library databases

Databases	Search strategy	Results	
Medline	Hydronephrosis AND Ureteral obstruction AND Vascular prosthesis OR vascular graft OR blood vessel prosthesis	papers found: 38 papers reported: 23	case reports: 11 case series: 4 retrospective studies: 5 prospective studies: 3
Embase	Hydronephrosis AND Ureteral obstruction AND Vascular prosthesis OR vascular graft OR blood vessel prosthesis	papers found: 52 papers reported: 14	case reports: 7 case series: 2 retrospective studies: 4 prospective studies: 1
Lilacs	Hydronephrosis	papers found: 161; papers reported: zero	
	Hidronefrose	papers found: 168; papers reported: zero	
	Ureteral obstruction	papers found: zero	
	Obstrução ureteral	papers found: 120; papers reported: zero	
Cochrane	Hydronephrosis	papers found: 124; papers reported: zero	
	Ureteral obstruction	papers found: 185; papers reported: zero	
Final results		27 papers	case reports: 13
			case series: 5
			retrospective studies: 6
			prospective studies: 3

Six retrospective^1,3-7^ studies attempted to estimate the incidence of this pathological condition, but the results were dissimilar. Wright et al.^1^ reported on 33 years of experience with 58 ureteral complications in 50 out of 3580 patients who had undergone aortoiliac reconstructive surgery. Just 42 patients presented hydronephrosis, thus revealing very low incidence. Gil-Salom et al.^4^ reported one case of hydronephrosis out of 50 aortobifemoral bypass procedures. However, Frusha et al.^5^ reported an incidence of 14% among 50 patients who underwent aortobifemoral bifurcation grafts, and Heard et al.^6^ noted incidence of 10% of the patients and 7% of the ureters in a study on 20 patients.

Three prospective studies^8-10^ attempted to discover the real incidence of this complication. Goldenberg et al.^8^ performed serial ultrasound examinations on 93 patients who had undergone aortofemoral or aortoiliac reconstructive surgery (one week, three months and one year postoperatively) and found that hydronephrosis developed in 11 patients (12%). The obstruction resolved spontaneously in 10 of these patients within three months and only a single case persisted for one year. In a similar study, Daune et al.^9^ reported no cases of symptomatic early or late hydronephrosis among 30 patients who underwent aortobifemoral graft. In another prospective study, Henriksen et al.^10^ did not find any patients with signs of ureteral obstruction among 56 patients who underwent aortic reconstruction.

After reviewing both the retrospective and the prospective studies, we could see that real urological complications following vascular grafts, such that surgical interventions were necessary, were described well in the case reports. We also discerned that well-designed studies are needed in order to validate the diagnosis and management.

Several pathogenic mechanisms have been suggested for the development of hydronephrosis. Early reports considered that placement of the graft anteriorly to the ureter, thus entrapping the ureter between the graft and the native artery, was the factor responsible. Other mechanisms that have been implicated include mechanical compression of the ureter by means of iliac or proximal anastomotic pseudoaneurysm, ureteral fibrosis due to constant microtrauma caused by graft pulsation, or direct injury to the ureter during surgery. However, it seems that in the majority of cases, the etiology of the obstruction is postoperative retroperitoneal fibrosis caused by tissue reaction to the implanted graft. The degree of the reaction correlates with the severity of surgical trauma and the residual hematoma.^11^

Treatment aims to restore urinary tract continuity and preserve kidney function. The type of therapy is chosen taking into account the type of lesion, time of occurrence, functional capacity of the corresponding kidney and patient status. A conservative approach is recommended for incidental asymptomatic cases of early postoperative hydronephrosis, since spontaneous resolution may occur in a high proportion of these patients.^8^ However, when symptoms are present or severe and late hydronephrosis is found, an operative procedure tends to be performed. The approaches used go from ureterolysis to nephrectomy. Ureterolysis, transection of the ureter and end-to-end anastomosis is an option of interest. Some authors prefer to divide and perform reanastomosis on the graft, in order to avoid opening the ureter and prevent extravasation of potentially infected urine and graft sepsis.^12^ In our opinion, it is a good alternative in cases of associated graft complications.

## CONCLUSION

Symptomatic ureterohydronephrosis following aortoiliac graft is a real complication and it needs to be quickly diagnosed and treated by urologists. The approach should be selected based on the particular features of each case.
